# Bayesian Integration and Non-Linear Feedback Control in a Full-Body Motor Task

**DOI:** 10.1371/journal.pcbi.1000629

**Published:** 2009-12-24

**Authors:** Ian H. Stevenson, Hugo L. Fernandes, Iris Vilares, Kunlin Wei, Konrad P. Körding

**Affiliations:** 1 Department of Physical Medicine and Rehabilitation, Northwestern University and Rehabilitation Institute of Chicago, Chicago, Illinois, United States of America; 2 Department of Physiology, Northwestern University, Chicago, Illinois, United States of America; 3 Department of Applied Mathematics, Northwestern University, Chicago, Illinois, United States of America; University College London, United Kingdom

## Abstract

A large number of experiments have asked to what degree human reaching movements can be understood as being close to optimal in a statistical sense. However, little is known about whether these principles are relevant for other classes of movements. Here we analyzed movement in a task that is similar to surfing or snowboarding. Human subjects stand on a force plate that measures their center of pressure. This center of pressure affects the acceleration of a cursor that is displayed in a noisy fashion (as a cloud of dots) on a projection screen while the subject is incentivized to keep the cursor close to a fixed position. We find that salient aspects of observed behavior are well-described by optimal control models where a Bayesian estimation model (Kalman filter) is combined with an optimal controller (either a Linear-Quadratic-Regulator or Bang-bang controller). We find evidence that subjects integrate information over time taking into account uncertainty. However, behavior in this continuous steering task appears to be a highly non-linear function of the visual feedback. While the nervous system appears to implement Bayes-like mechanisms for a full-body, dynamic task, it may additionally take into account the specific costs and constraints of the task.

## Author Summary

There is a growing body of work demonstrating that humans are close to statistically optimal in both their perception of the world and their actions on it. That is, we seem to combine information from our sensors with the constraints and costs of moving to minimize our errors and effort. Most of the evidence for this type of behavior comes from tasks such as reaching in a small workspace or standing on a force plate passively viewing a stimulus. Although humans appear to be near-optimal for these tasks, it is not clear whether the theory holds for other tasks. Here we introduce a full-body, goal-directed task similar to surfing or snowboarding where subjects steer a cursor with their center of pressure. We find that subjects respond to sensory uncertainty near-optimally in this task, but their behavior is highly non-linear. This suggests that the computations performed by the nervous system may take into account a more complicated set of costs and constraints than previously supposed.

## Introduction

Recent studies have shown that, for many motor tasks, human subjects take uncertainty in their sensory feedback into account. They often use knowledge of uncertainty in a way that is close to optimal in a statistical sense both in their perception of the world [c.f. 1],[2],[3] and for several types of movement [4]–[7]. Subjects' behavior is accurately predicted by normative models that describe what we “should” do given uncertainty arising from noisy sensory information and constraints on action [8]. The focus of the majority of these normative models is Bayesian statistics, which describes how different pieces of uncertain information should be combined. For instance, given cues from two noisy sensors Bayesian statistics predicts that an ideal observer would combine information from the two sensors weighted by the precision of each sensor [1],[9]. There is growing evidence that the nervous system may implement these types of Bayesian computations [10]–[18]. However, most of this evidence is based on studies of pure perceptual judgment or relatively simple behaviors such as hand-reaching. They generally do not address dynamical aspects of movement control or the unconstrained movements that we use in daily life. The control of these movements requires the nervous system to extract relevant information from a rapidly changing, noisy environment and to coordinate multiple effectors. A central question for our understanding of the computations the brain performs is whether uncertainty still plays a role during coordinated, full-body sensorimotor tasks.

In studies of Bayesian behavior, the problem of how the brain uses sensory estimates to control movement has often been formulated as an optimization problem. That is, given the constraints and costs of the movement as well as sensory information, the nervous system computes how to move to minimize the cost. A range of human movement studies have been conducted confirming that humans often move in a way that is close to statistically optimal, in this sense [19]–[26]. Subjects appear to estimate the state of the world conforming to Bayesian mechanisms - combining information across sensors and time in a way that takes uncertainty into account, and subjects appear to move to minimize cost functions that quantify their performance error and control effort. For instance, errors between hand position and a target or between current posture and standing upright seem to be penalized with the square of the error [20],[24]. These studies based on optimal control have advanced our understanding of basic human behavior, but it is not yet clear how accurate these descriptions will be for more complex behaviors. Here we attempt to generalize these theories to a continuous, full-body task.

We introduce a new goal-directed, visuomotor task where whole-body movements are required to interact with the environment. In this task subjects steer a noisy, dynamic visual cursor by forward-backward shifts of body weight similar to surfing or snowboarding. Our purposes are two-fold. First, we aim to test whether Bayesian predictions of the behavioral responses to visual feedback still hold when the task dynamics are more complex. Second, we aim to test whether, as in studies of reaching and quiet standing, subjects appear to use a linear feedback control rule with a quadratic cost function. We find that many aspects of behavior are well captured by optimal control models incorporating Bayesian estimation of feedback uncertainty. However, behavior during this task differs in an important way from previous work on simple movements such as hand reaching and quiet standing. In this steering task human subjects appear to combine two well-known control strategies: bang-bang control and linear-quadratic regulation. Importantly, our results suggest that humans still take uncertainty into account during a full-body, dynamical control task.

## Materials and Methods

### Ethics statement

All experimental protocols were approved by IRB and in accordance with Northwestern University's policy statement on the use of humans in experiments. Informed consent was obtained from all participants.

### Experimental details

**Figure 1:**
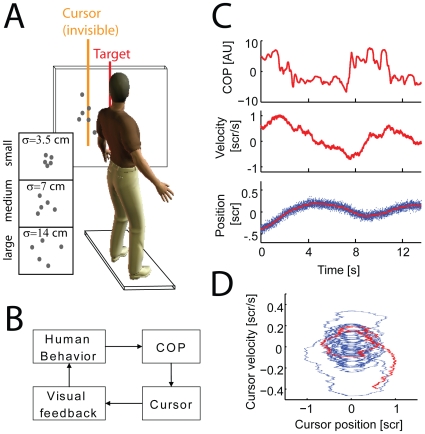
The task and data. A) The experimental setup. Subjects steer a cursor by shifts in center of pressure (COP) along the anterior-posterior axis. Noisy feedback of the cursor position (small, medium or large variance) is given while subjects are incentivized to steer the cursor to be close to the midline of the screen (target). B) Subject's movements affect the center of pressure, which is measured by a force plate. The resulting sensor readings then steer the on-screen cursor. Subjects receive noisy visual feedback about the cursor position and react to reduce errors. C) COP, cursor velocity and cursor position are shown as a function of time during one trial for a typical subject (red). The observed feedback (noisy dots) is shown in blue. D) The phase portrait of cursor position and velocity is shown for 10 successive trials. Data from (C) are highlighted in red.

Here we use a novel approach to analyze the influence of uncertainty on the dynamical control of subject's movement (see Fig. 1A and B). In this experiment a force plate measures the movement of subject's center of pressure (COP). This COP dynamically steers the movements of a cursor on the screen and visual feedback about the cursor position is corrupted by noise. To analyze the effect of feedback uncertainty we vary the quality of feedback between low, medium or high uncertainty from trial to trial. Due to process noise in the dynamics of the cursor, human subjects have the task of stabilizing the cursor near the center of the screen in the presence of ongoing fluctuations. Subjects receive monetary rewards for successful stabilization.

The goal of this experiment is to examine how subjects control a noisy dynamical system during a goal-directed, full-body steering task. 10 healthy volunteers participated in the experiment. (4 female, 6 male; age 30.7 ± 5.0 years; weight 67.6 ± 8.3 kg). Subjects were instructed to stand perpendicular to a rear-projection screen (1.41 m ×0.79 m), ∼0.6m away, on a 4-sensor force-plate (Nintendo Wii Balance Board, recorded at 500 Hz) (see Fig. 1). By moving their body, subjects could control the acceleration of the cursor through their center of pressure (COP) along the anterior-posterior axis with the dynamics of the cursor following:
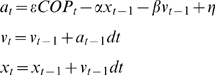
where 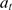 represents the acceleration, 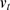 the velocity, and 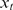 the position of the cursor at time 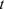. Subjects influence the cursor through 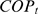 (the subject's A-P center of pressure in cm), and 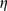 represents process noise which follows 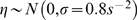. Finally, 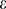 parameterizes the influence the subject has on the cursor, and 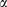 and 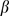 are parameters preventing the cursor from going too far off-screen. Normalizing by the screen-size, we chose 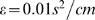, 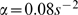 and 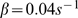. With these dynamics, controlling the cursor is quite difficult, and large errors in cursor position are relatively frequent. The observed standard deviation of the cursor position is ∼0.18 scr, where scr denotes screen units which range from [−0.5,0.5]. Depending on their preference, 8 subjects faced the screen with their left foot forward (called regular in the surfing community) and 2 subjects with their right foot (goofy).

The experiment was divided into 180 trials with each trial lasting for a random duration evenly distributed between 11.5 and 15 seconds. Every 20 ms a new dot with low contrast was shown on the screen with a position drawn from a radially isotropic Gaussian distribution centered on the true position of the cursor, while the previously shown dot disappeared. Due to persistence of vision, subjects perceive a rapidly fluctuating cloud of ∼5–10 dots. The width of this Gaussian cloud changed randomly from trial to trial with three categories: small, medium, or large variance (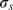 = 3.5 cm, 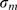 = 7 cm and 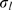 = 14 cm).

At the end of each trial the true cursor position was revealed. Subjects were subsequently given a score based on the squared distance between the cursor and the mid-line of the display. The random trial duration incentivizes subjects to minimize the error over the entire trial, not simply the final error. The monetary rewards were arranged such that the minimum reward obtainable over the course of the experiment was $$ 10 and the maximal reward obtainable was $$ 20.

To account for the possibility that the cursor dynamics in this task cause subjects to approach biomechanical limits and behave atypically, we ran a similar experiment (N = 5, 1 female, 4 male, separate from the original group) in which the control gain was increased by a factor of four (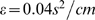). This high-gain condition makes the task substantially easier. In this case subjects make much smaller errors (standard deviation of the cursor position ∼0.16 scr), and the task requires a much smaller COP range (standard deviation of 2.96 cm compared to 5.07 cm in the original experiment).

The cursor dynamics in this task are based on a stochastic linear dynamical system, where the state of the world evolves linearly with some process noise and subjects receive noisy feedback. Uncertainty arises from both the state evolution, through the process noise 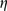, and the feedback, through the observation noise 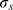, 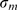, or 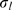. In the sections that follow, we briefly present the ideal observer model (the Kalman filter) that allows optimal state estimation for this system and the optimal control models that describe what action an ideal observer should take given their state estimates and the costs of specific actions.

### Control models

We compare four different models of behavior for this task. Our objective is to predict subject's center of pressure 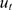 based on their observations, i.e. the noisy position 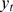 of the dots on the screen. The first model, a proportional-integral-derivative controller (PID), simply uses these observations directly. The second two models assume an ideal observer (Kalman Filter) and estimate the control under different cost assumptions: quadratic costs (linear-quadratic regulator - LQR) and negligible costs in a small, fixed range of control (bang-bang controller). Finally, we consider a non-linear extension of the LQR controller. For all models we fit the parameters by minimizing the squared distance between measured and predicted COP trajectories: 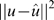.

In model 1, the proportional-integral-derivative controller (PID), we assume that the observer ignores the dynamics of the cursor and simply estimates the best policy based on the noisy observations 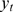:
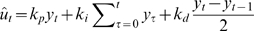


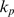
, 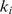, and 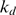 parameterize the contributions of the proportional, integral, and derivative terms respectively. PID controllers have previously been used to explain human postural control [26],[27], and while this model does not explicitly estimate the underlying position of the cursor, the integral term allows fluctuations in the feedback noise to be averaged over time.

In models 2 and 3 we use a standard Kalman filter to compute the estimated state of the cursor from the observations [28]. The Kalman filter assumes that the state 
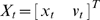
 of the cursor at time *t* evolves from the state at time *t-1* according to linear dynamics and control: 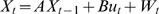. Here 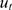 is the control signal used by the system and 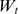 is process noise drawn from a Gaussian distribution. We assume an ideal observer that has full knowledge of the dynamics *A*, the effect of control *B*, and the distribution of 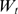 used during the experiment. In this case, *A* and *B* follow immediately from the set of difference equations used to control the cursor (see Experimental details) and 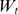 reflects the fluctuations in acceleration or process noise 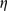.

An important feature of the Kalman filter as it relates to this experiment is how estimation changes as function of feedback uncertainty. The best estimate of the state at time *t* combines the *a priori* state estimate (from *t-1*) with the current observation. Increasing the observation noise (feedback uncertainty) while keeping the dynamics and process noise the same causes the observation to have a smaller effect on how the current state estimate is updated (the Kalman update). That is, as feedback uncertainty increases the observations have a weaker effect and are integrated more slowly over time.

The following models use the Kalman filter state estimates. However, to be optimal we must define an underlying cost function, which will determine the control policy. In model 2 we consider a linear-quadratic regulator [20]. Following the actual rewards during the task, this control policy minimizes the squared end-point error as well as the control itself with the cost function 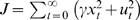. In this particular case, 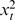 penalizes how far the cursor is from the target and 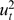 penalizes deviations from upright posture (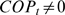). Here 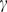 balances how lazy subjects are in comparison to how badly they want to perform well. The solution *K* to the matrix Riccati equation minimizes the above cost function, and yields a simple rule which corresponds to the linear feedback control
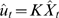



To fit the free parameters, we optimize over 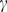 and the feedback uncertainty for each of the three feedback conditions (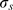, 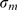, and 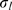) to fit human behavior. The model thus has 4 free parameters. Note that, in the experiment, monetary rewards are given proportional to the squared error at the end of each trial rather than continuously. However, minimizing the error term in the cost function *J* over all time will maximize the monetary reward function as well, since the real rewards are presented at pseudo-random times.

Model 3 again uses an ideal observer; however, here we assume that subjects use another type of control policy: a bang-bang controller. This model assumes two-state control with a threshold determined by a combination of the estimated position and velocity:
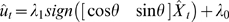



Here 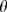 parameterizes the decision rule for a given position and velocity, and 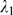 and 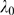 parameterize the magnitude of the two states of the bang-bang controller. If control costs are negligible in comparison to the rewards but the control signal is limited - either because subjects do not want to fall of the board or due to biomechanical constraints - then this control scheme is actually optimal.

Finally, in model 4, we consider a non-linear extension of the linear-quadratic regulator. This model estimates the optimal control for a standard linear-quadratic regulator. Then, to approximate the constraints of human behavior during this task (not wanting to fall over or biomechanical limits on posture), we pass the control predicted by the linear-quadratic regulator through a static non-linearity (a logistic function). Although this control scheme is sub-optimal for the two classes of cost-functions we consider in models 2 and 3, the static non-linearity serves to interpolate between bang-bang control and LQR. Bang-bang control is limited in the sense that it must explain a continuous signal using only two states, and LQR is limited in that it does not appropriately model the constraints and costs of the task, such as not wanting to fall off the board.

## Results

### The effects of feedback uncertainty

**Figure 2:**
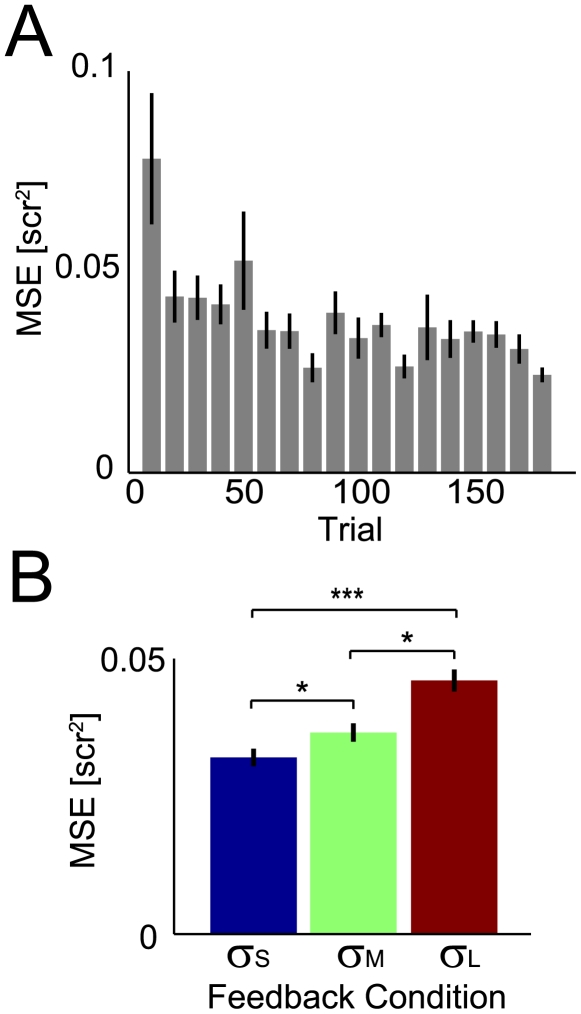
Task errors across time and across feedback conditions. A) Average errors across subjects over the course of the experiment binned in blocks of 10 trials. B) The influence of feedback type on task errors. All comparisons between feedback uncertainty levels were significant (one-sided t-test). In both plots errorbars denote SEM across 10 subjects. * denotes p<0.05. *** denotes p<0.001.

We find that human subjects readily learn our task. While the noise introduced into the cursor dynamics constantly perturbs the movement of the cursor, subjects are able to change their COP and stabilize the cursor position (see Fig. 1C). The dynamics of the cursor induce weak oscillations in the cursor position and humans readily dampen this behavior (see Fig. 1D). Subjects show quick improvement over the first couple of trials but continue to improve slowly over the course of 180 trials (Fig. 2A). Several subjects reported that controlling the cursor was difficult, and subjects make large deviations from upright posture throughout the experiment.

In trials where the feedback is better human subjects have lower mean squared errors (MSEs) on average (Fig. 2B). This is consistent with a number of previous experiments and can be explained by estimation errors alone. However, we can also examine the specific strategies human subjects use to deal with the continuous nature of the task.

**Figure 3:**
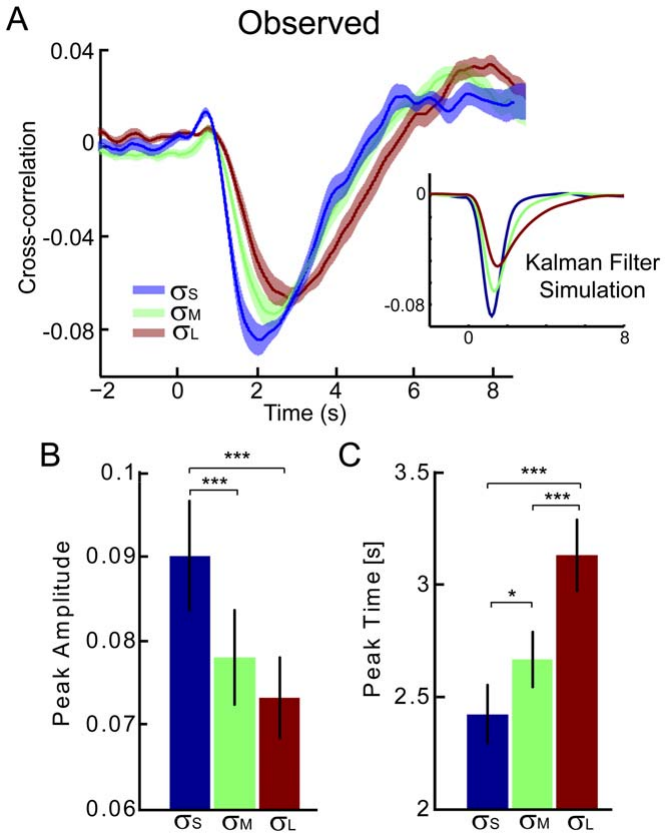
Cross-correlations between process noise and COP. A) Cross-correlation between the fluctuations in cursor acceleration (process noise, 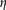) and the center of pressure with time lag for each feedback uncertainty level. The inset shows the cross-correlation between fluctuations in the cursor acceleration and the Kalman update in a simulation. The results have been smoothed to mimic postural responses (Gaussian smoothing, 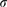 = 250 ms) B) Peak amplitude for each feedback uncertainty level. C) Peak time for each feedback uncertainty level. Confidence intervals denote SEM (N = 10). * denotes p<0.05; *** denotes p<0.001 (one-sided paired t-test).

One direct way of analyzing the behavior in this task is to observe subjects' responses to fluctuations in the time domain. Taking the cross-correlation between the fluctuations in cursor dynamics (process noise, 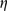) and the center of pressure we find that responses to fluctuations in cursor position are consistent with ideal observer models. That is, we find that subjects respond more slowly and with lower amplitudes when the feedback is more uncertain (Fig. 3A). Peak response amplitude to small uncertainty feedback was significantly higher than in the other two feedback conditions (p<0.001 for both comparisons, one-sided paired t-test). In addition, the peak response time was significantly different across all feedback conditions (p<0.05 for all comparisons, one-sided paired t-test, Fig. 3B), with higher feedback uncertainty corresponding to slower responses. Feedback uncertainty is significant as a main effect for both peak time and peak amplitude (single factor, repeated measures ANOVA, p = 0.000035 and p = 0.00095 respectively). While there is a large variability across subjects, the ordering of peak time and amplitude within subjects is highly stereotyped with larger feedback uncertainty being associated with slower, weaker responses.

These results are qualitatively predicted by the Kalman filter models, since the Kalman update decreases with increasing feedback uncertainty. Small Kalman updates then lead to longer integration times and smaller excursions. For reference we include results from a simulation showing the cross-correlation between fluctuations and the Kalman update for three levels of feedback uncertainty (Fig. 3A inset). In these simulations the control 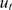 was fixed at zero. Since the Kalman filter performs estimation alone, changes in the Kalman update occur immediately after fluctuations and the cross-correlation decays approximately as an exponential. The observed cross-correlations, on the other hand, are based on subject's actions and are only an indirect reflection of subject's state estimates. The shape of the observed cross-correlations is consistent with simulation results that have been phase lagged and low-pass filtered. For comparison we have low-pass filtered the simulation results (Gaussian smoothing, 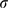 = 250 ms).

The focus of the high-gain experiment is whether the range of center of pressure required for the task affects subject's control strategies. We do not expect any qualitative differences in how subjects estimate the cursor position. Indeed, we find similar trends for the case where the control gain is much larger. For the 5 subjects in the high-gain condition, the mean-squared target errors are 0.022±0.007 scr^2^, 0.027±0.007 scr^2^, and 0.054±0.016 scr^2^ for 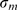, 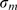, and 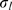 respectively. We again see that subjects show quick improvement over the first couple of trials and continue to improve slowly over the course of the experiment. Mean cross-correlation amplitudes are 0.048±0.006, 0.047±0.005, and 0.038±0.006 for 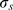, 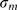, and 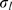 respectively, and mean cross-correlation peak times are 2.22±0.14 s, 2.43±0.09 s, and 2.83±0.31 s for 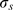, 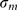, and 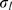. As before, these results are consistent with an ideal observer model integrating information more slowly as feedback uncertainty increases.

It is important to note that the predictions of the ideal observer model (Kalman filter) describe perception alone. Since we measure postural responses, the above analyses serve as indirect evidence for near-optimal Bayesian integration. However, the ordering of peak time and peak amplitude responses clearly indicates that subjects take feedback uncertainty into account. Moreover, this ordering is consistent with an ideal observer using a monotonic feedback control rule,

### Estimating control policies and model comparison

**Figure 4:**
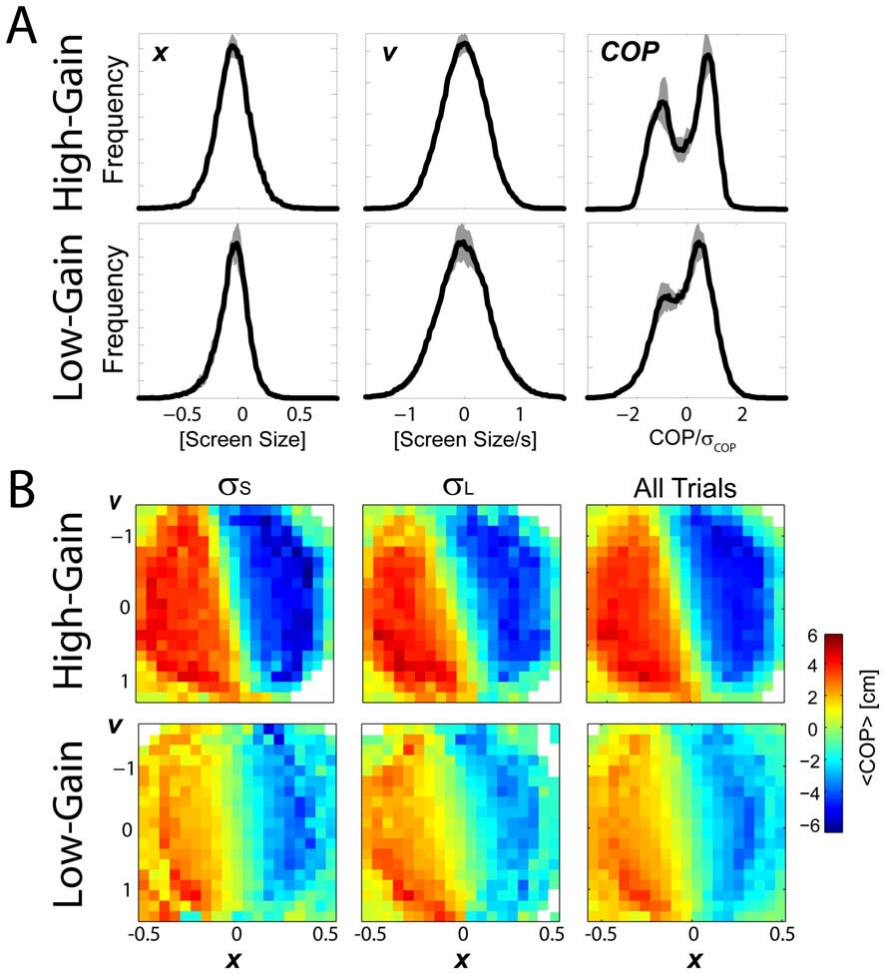
Control policies. A) Distributions of the cursor position (left), cursor velocity (center) and center of pressure normalized by the standard deviation (right), averaged across subjects for the low-gain (top row) and high-gain (bottom row) conditions. In the low-gain condition, note the bimodal distribution of the center of pressure, despite the unimodal distribution of errors. This may indicate a bang-bang-like strategy. B) Policy-maps of the center of pressure averaged across subjects as a function of the true cursor position and velocity for two different levels of feedback uncertainty and across all conditions. Note that in the low-gain condition subject's responses saturate at large cursor velocities and positions. In the high-gain condition responses are much more linear.

Although subjects respond differently to different types of feedback, we can also look in detail at the strategies subjects used during the task – their control policies. To do this we compute the average center of pressure (the response) given the true cursor position and cursor velocity (the state) for each of feedback condition (Fig. 4B). Given the state of the cursor, the policies illustrate the control issued by subjects. In stark contrast to previous reaching experiments, we find that subjects' control policies appear qualitatively more similar to bang-bang controllers than to linear-quadratic-regulators (Fig. 4B, top row). Instead of a plane in the space of positions and velocities, center of pressure appears to saturate at large velocities and positions. The distribution of center of pressure averaged across subjects (Fig. 4A, top right) also suggests a type of approximate two-state control. Subjects tend to lean fully forward or fully backward despite the fact that errors in cursor position are unimodally distributed.

This non-linear control strategy may be due to the wide range of center of pressures required for the task. In the high-gain condition, where center of pressure excursions can be much smaller for a given error level, subject's behavior appears much more linear. The COP distribution appears more unimodal (Fig. 4A, bottom right), and subject's control policies are qualitatively much more similar to a plane than a saturating non-linearity. Nonlinear control still occurs, however, for cases where large center of pressure excursions are helpful for performing the task and may be a result of postural biomechanics far away from upright standing.

**Figure 5:**
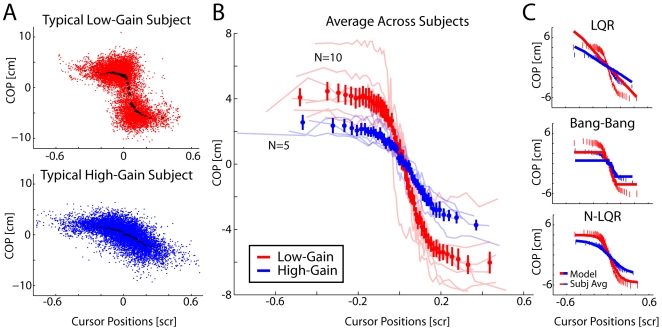
Policies as a function of position. A) Center of pressure as a function of cursor position for typical subjects in the low and high-gain conditions. Black lines denote median responses for a given range of cursor positions. Red and blue points denote samples along the COP trajectory. B) Average responses across subjects with thin lines denoting the responses of individual subjects. C) The predicted responses from the LQR, Bang-bang, and Non-linear LQR models. Error bars denote SEM across subjects (in B and C) and sample points (in A).

We also examined how subject's controlled their center of pressure as a function of the cursor position alone (Fig. 5). These analyses highlight the non-linearity of the control policies and the differences between the low-gain and high-gain tasks. Both individual subjects (Fig. 5A) and the across subject average (Fig. 5B) show highly non-linear behavior in the low-gain condition and much more linear behavior in the high-gain condition.

**Figure 6:**
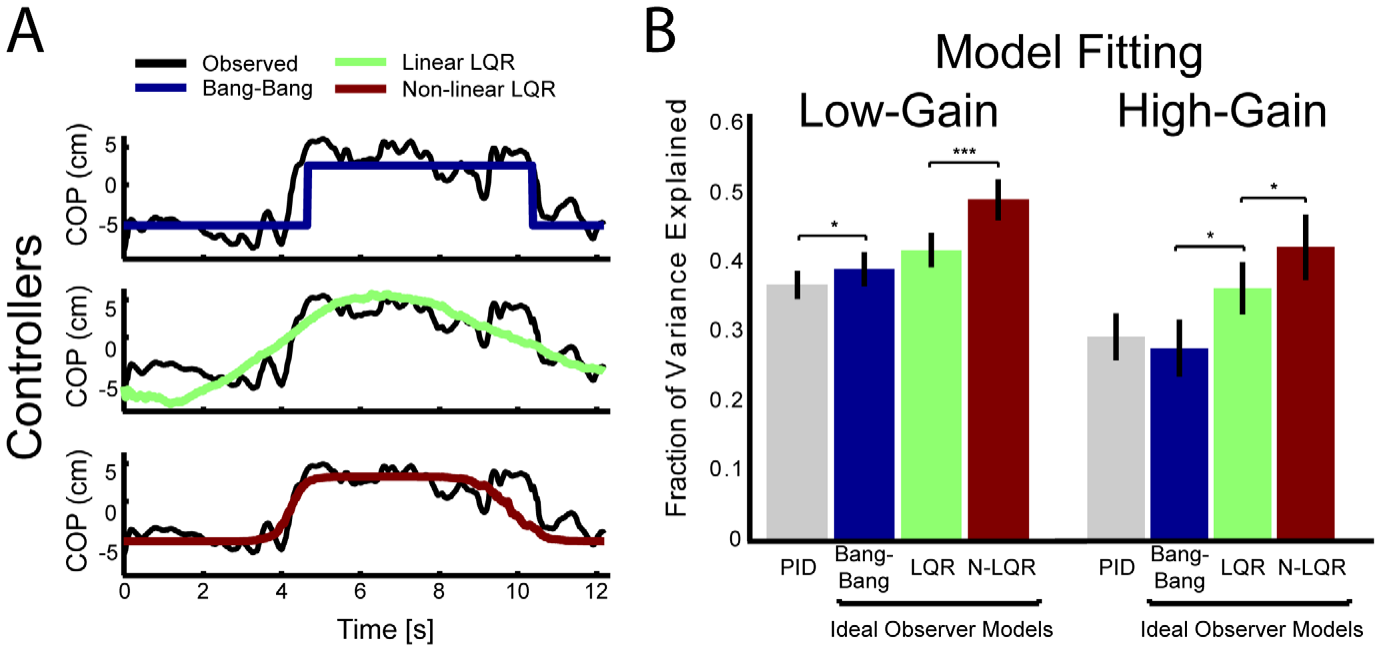
Model fitting. A) Observed center of pressure for a typical subject and trial along with the center of pressure predicted by each of the three ideal observer models. Note that the linear-quadratic-regulator and the bang-bang controllers produce qualitatively very different estimates. Note also that the non-linear LQR model has some ability to interpolate between the two. B) Cross-validated fraction of variance explained for each model for both the low and high-gain experiments (two-fold cross validation). In the low-gain condition the ideal optimal observer models explain a significantly larger fraction of variance than the PID controller (p<0.05, one-sided paired t-test), and the non-linear LQR explains a significantly larger fraction of variance than all others (p<0.001, one-sided paired t-test). Error bars denote SEM across subjects.

The bang-bang controller appears qualitatively very similar to human behavior (Fig. 4A–B). To quantify this similarity we fit each of the four models above (see Materials and Methods) to the behavior of individual subjects. Model 1, the PID controller, provides a first approximation of human behavior during this task. It is not particularly surprising that this model does not fit well, since the observed behavior appears very non-linear and the model does not take into account the cursor dynamics. The three ideal observer models (models 2–4) all explain significantly more variance than the PID model (Fig. 6B).

Model 2, the bang-bang controller captures the bimodal strategy observed in human behavior but is limited by the fact that it attempts to model a continuous signal using only two discrete states (Fig. 5C, Fig. 6A). Model 3, the standard LQR fails to capture the bimodal control strategy used by subjects: the predicted COP follows a unimodal distribution that reflects the distribution of errors and does not follow the non-linearity in subject's policies (Fig. 5C). Although the standard LQR model uses a PD controller (linear control based on position and velocity), the addition of a state estimation model (Kalman filter) confers some advantage over the controllers based on the observations alone, such as the PID controller (Fig. 6B). Not including dynamic state estimation reduces the fraction of variance explained by ∼8% (9.1% for LQR, 7.7% for the Bang-bang controller). Using state estimation but without including the cursor dynamics reduces the fraction of variance explained by ∼4% (4.5% for LQR, 4.3% for Bang-bang).

Finally, by combining aspects of the bang-bang and standard LQR controllers, a non-linear LQR model (model 4) out-performs all other models. This model captures the continuous character of the signal, and also allows for saturation-like effects where the nature of the task constrains behavior (Fig. 5C). All models were fit after throwing out the first 20 trials to remove initial learning effects.

It should be noted that Figure 6 shows the cross-validated fraction of variance explained. The models were fit on one half of the data (odd trials), while prediction error was estimated from the second half of data (even trials). Since the four models have different numbers of free parameters (PID: 3, Bang-bang: 6, LQR: 4, NLQR: 7), differences in the prediction error on training data may be due to over-fitting. However, in the results presented cross-validation controls for these differences in model complexity.

## Discussion

Here we have shown that ideal observer and optimal control models can describe many aspects of human behavior in a surfing-like task where movements of the body steer the movements of a cursor. We have found that there is a clear influence of uncertainty on motor behavior. As predicted by Bayesian statistics (Kalman filter model), subjects respond more slowly and with lower amplitude to higher uncertainty feedback suggesting that they are integrating information over longer periods of time. Unlike previous (predominantly reaching) experiments examining the effects of uncertainty on behavior, we find that under certain conditions subjects use highly non-linear strategies similar to bang-bang control. These results suggest that human subjects take the uncertainty of sensory information into account and use this information during motor control, even during full-body behavior when the task is continuous and constrained by biomechanical factors.

Several studies have examined behavior during tasks involving control of the center of pressure including skiing on a simulator [29],[30], snowboarding in a virtual reality setting [31], and rocking the body on a force plate [32]. However, these studies mostly address motor learning questions without addressing control or uncertainty. In the task presented here we varied uncertainty parametrically and subjects performed an explicitly goal-driven task. While many reaching tasks also examine these effects, here we use a continuous task with constrained control signals, limited by the support surface.

The present study provides strong evidence that feedback uncertainty affects online control of continuous movements. When feedback is more uncertain the behavioral responses are significantly slower, indicating the nervous system needs to integrate information over a longer period of time. Similar results have been reported for reaching tasks where reaction time increases with increasing uncertainty about the target [33]. When a target is perturbed visually, adaptation to the perturbation is also slower when there is more visual uncertainty associated with the target representation [33],[34]. All these findings are in accordance with Bayesian models of sensory estimation. Our study highlights the effect uncertain information has on online, continuous control in complex motor tasks other than the well studied point-to-point reaching task.

Previous studies of optimal control in reaching have found that human behavior is accurately modeled by linear-quadratic regulation [35]. Muscle activations in response to support surface perturbations also appear to be well-described by near-optimal linear feedback rules [36]. Here we find that, for certain tasks, human behavior appears to be highly non-linear. This deviation from previous models may be due to the particular properties of our task, where control signals are limited in size by costs (subjects cannot afford to fall of the force plate) or biomechanical factors. At the same time, when posture is close to upright, the task is characterized by relatively low control costs. In the high-gain condition, where the distribution of center of pressures required for the task is much smaller, we find that behavior is much more linear. Only when body postures get toward extreme values do biomechanics and a risk of falling off induce constraints on behavior.

The models presented here aim to describe the factors that drive motor control in dynamical situations. However, unlike in reaching tasks where two-link systems provide fairly accurate biomechanical models, the experiment here needed to be simplified dramatically to allow for productive modeling. Specifically, we ignore the biomechanical factors that link the motor commands driving body stabilization with actual movements of the center of pressure. This simplifying assumption makes modeling much more tractable but could potentially be extended with more realistic biomechanics. We should note, however, that the dynamics of the body should have a small effect on the results presented here. Although the natural frequency of quiet standing is on the order of one second [37], reaction time (from a sensory stimulus to a change in the center of pressure) is on the order of 100 s of milliseconds [38]. Changes to the cursor position and in subjects' posture thus occur on a slower timescale than the timescale of possible posture responses.

Despite this difference in timescales, the cursor dynamics in the low-gain condition apparently do cause subjects to use the full range of their center of pressure, allowing us to observe control strategies near the biomechanical limits of posture. The high-gain experiment was designed to make the task much easier and requires subjects to use a much smaller range of postures. In this case, subjects use much more linear control strategies. Importantly, both these regimes, near equilibrium and near biomechanical limits, exist in normal human behavior, and appear to be well-described by control models that use optimal state estimation. We should also note that, for the results presented here, the problem of how subjects estimate the cursor position is inter-twined with the problem of how subjects control the cursor. The timescales of estimation alone are likely to be faster than those shown.

In addition to computational implications, the results presented above may also have implications for neurophysiological studies. In the past decade several studies have made progress investigating the neural correlates of uncertainty and Bayesian computations [10], [12], [13], [39]–[42]. Several lines of research suggest that feedback uncertainty is represented in both pre-motor and medial temporal cortex during sensorimotor tasks [15]–[18], and that movement errors are represented in cerebellum [43],[44]. The results presented here suggest that the nervous system represents feedback uncertainty continuously and dynamically and is able to integrate feedback uncertainty over time. The control policies we observe suggest that the output of the nervous system may be nonlinear; however, this nonlinearity may be due to biomechanical factors. As such, this experiment does not rule out the possibility that cerebellar error computations may be linear.

Here we have combined aspects of typical experiments that ask if the nervous system employs Bayesian strategies with aspects of typical experiments that analyze the dynamical control of movements. We have found that salient aspects of optimal control and optimal Bayesian estimation can be observed for a complex task where whole-body movements are controlled continuously. This may indicate that these principles describe general properties of the human movement system and that people can rapidly learn to control a system in a near-optimal way – even if a non-linear control scheme such as bang-bang-like control is necessary.
